# Comparison of the Acute Effects of Kinesio Taping and Sleeper Stretching on the Shoulder Rotation Range of Motion, Manual Muscle Strength, and Sub-Acromial Space in Pitchers with Glenohumeral Internal Rotation Deficit

**DOI:** 10.3390/medicina57020102

**Published:** 2021-01-23

**Authors:** Chi-Ling Lo, Ya-Hsin Hsueh, Chun-Hou Wang, Hsiao-Yun Chang

**Affiliations:** 1 Department of Physical Medicine, Cheng Ching General Hospital, Taichung 40764, Taiwan; chulingfit@gmail.com; 2 Department of Electronic Engineering, National Yunlin University of Science and Technology, Yunlin 640301, Taiwan; hsuehyh@yuntech.edu.tw; 3 Department of Physical Therapy, Chung Shan Medical University, Taichung 40201, Taiwan; chwang@csmu.edu.tw; 4 Physical Therapy Room, Chung Shan Medical University Hospital, Taichung 40201, Taiwan; 5 Department of Athletic Training and Health, National Taiwan Sports University, Taoyuan 333325, Taiwan

**Keywords:** stretch, posterior shoulder tightness, overhead sports, elastic taping, shoulder injury

## Abstract

Background and Objectives: Sleeper stretching (SS) can improve the shoulder’s range of motion (ROM) for pitchers with glenohumeral internal rotation deficit (GIRD). However, no evidence has proven the effect of Kinesio taping (KT) on shoulder strength and ROM. Therefore, this study compared the effects of SS and KT on shoulder rotation ROM, muscle strength, and sub-acromial distance in pitchers with GIRD. Materials and Methods: Thirty-one pitchers with GIRD were allocated into control, KT, and SS groups. Shoulder rotation ROM, muscle strength, and sub-acromial space were measured before and after treatment with SS or KT. Results: The results revealed that KT and SS significantly enhanced shoulder rotation ROM in pitchers with GIRD. External rotator strength significantly increased following KT but significantly decreased after SS. KT and SS exerted no effects on the sub-acromial space. Conclusions: KT and SS improve shoulder rotation ROM in pitchers with GIRD. In addition, KT improves shoulder external rotator strength, and SS reduces it.

## 1. Introduction

Shoulder injuries are extremely common injuries in baseball, with an average of 2.14–2.27 injuries per 1000 h of exercise [1]. These injuries mostly include shoulder impingement, rotator cuff injury, and labral tear, all of which cause pain in athletes’ shoulders and result in the inability to compete or train. The risk factors for shoulder injuries among pitchers are insufficient shoulder mobility and muscle strength [2,3]. In terms of insufficient shoulder mobility, repeated pitching increases the shoulder’s external rotation and reduces the range of internal rotation, leading to a glenohumeral internal rotation deficit (GIRD) [4]. In terms of insufficient muscle strength around the shoulder, imbalance in the strength of rotator muscles was noted in previous studies, especially weakness of the shoulder’s external rotators. This also causes an increased incidence of throwing injuries among pitchers.

In recent years, many researchers have highlighted the causes of shoulder injuries among pitchers, particularly those with GIRD, and have proposed many treatment methods. The sleeper stretch (SS) and the cross-body stretch are the most common methods used by pitchers to increase the range of internal rotation of the shoulder. Most related studies have observed improvements in internal rotation and horizontal adduction in athletes who use cross-body stretching and SS [5,6,7,8]. However, such studies only evaluated shoulder range of motion (ROM) and have not assessed the effect of stretching on muscle strength. In addition, previous studies have observed that static stretching before exercise may reduce muscle strength [9,10]. This is very disadvantageous for pitchers who are going to throw.

Previous studies have shown that taping can reduce pain in individuals with shoulder injuries while increasing ROM, sub-acromial distance, and muscle strength [11,12,13,14]. However, taping has various types and involves different methods, such as Kinesio taping (KT) and non-elastic taping. KT is an elastic tape that is commonly used in sports, and it can relieve muscle tightness, increase flexibility, and enhance proprioception [15,16]. This tape has permeability, ductility, and suitable adhesiveness, and it is thin and non-latex to minimize the likelihood of users having allergic reactions [17,18]. Şimşek et al. [14] divided 38 participants with sub-acromial impingement syndrome into two groups that were treated with sham taping and KT to evaluate their shoulder pain, ROM, and muscle strength. The KT was applied to the supraspinatus, deltoid muscles, and the acromion. The results indicated that KT could relieve shoulder pain and increase the shoulder’s flexion and abduction angles, and external rotation strength. However, in most of these studies, Kinesio tape was mostly applied to the deltoid, supraspinatus, and lower trapezius, to focus on treating sub-acromial impingement syndrome, and not GIRD or posterior shoulder tightness, for which the tape should be applied to the posterior deltoid, teres minor, or infraspinatus. Therefore, the purpose of this study was to compare the effects of SS and KT on shoulder ROM, rotator muscle strength, and sub-acromial distance among pitchers with GIRD.

## 2. Materials and Methods

### 2.1. Study Design and Sample Size Estimation

This study employed a pre–post test, comparative experimental design that divided pitchers with GIRD into three groups (control, SS, and KT group). The power and sample size calculation were done with G*Power Version 3.0.10 (Franz Faul, Universität Kiel, Düsseldorf, Germany). The researchers performed sample size calculations based on the following parameters: a 2-side test, power of 0.8, and α = 0.05. The main outcome measure was the ROM of shoulder internal rotation. Based on the results of a previous study [13] and mid-term results statistics, the researchers needed at least 10 subjects in each group.

### 2.2. Participants

This study recruited 31 pitchers with GIRD. Before the experiment, the participants received an explanation of the procedure from the researchers and signed a consent form. The ROM of shoulder internal rotation (IR) in their dominant arms and non-dominant arms were then measured at the beginning of the test. Players were considered to have GIRD if the reduction in the range of shoulder IR was greater than 10% of the total rotation for shoulder internal–external rotation; such players were recruited [4]. After recruitment, each participant was randomly assigned to the KT, SS, or control group through lot drawing. The others inclusion criteria for this study were as follows: more than 5 years of regular baseball training; frequent use of overhead throwing; and no shoulder injuries within 1 year before completing the experiment. The exclusion criteria were as follows: prior history of upper limb surgery; ongoing treatment or rehabilitation; and no use of overhead throwing. All subjects gave their informed consent for inclusion before they participated in the study. The study was conducted in accordance with the Declaration of Helsinki. The recruitment and study procedure were conducted after review and approval from the Institutional Review Board of Cheng Ching General Hospital, Taiwan (Project identification code: HP140039).

### 2.3. Procedures

**Figure 1:**
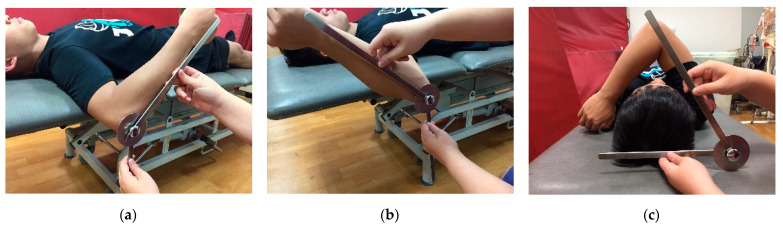
Measurements of a shoulder’s range of motion: (**a**) internal rotation, (**b**) external rotation, (**c**) horizontal adduction.

This study used the Baseline Goniometer Set (Sammons Preston Rolyan, Patterson Medical Products, Inc., Warrenville, IL, USA) to assess active shoulder ROM, which was measured three times; the average was used as the final value for statistical calculations. Shoulder ROM comprised shoulder internal rotation (IR), external rotation (ER), and horizontal adduction (HA), and total rotation calculated from internal-external rotation (Figure 1). In this study, ROM measurement referenced the standard postures mentioned by Norkin and Whire [19] with the ROM of IR and ER indicating the flexibility of the shoulder rotator cuff. The ROM for HA indicates the extent of posterior shoulder tightness; muscles that tighten easily include the posterior deltoid and middle trapezius [20]. For the shoulder ROM test for IR, ER, and HA, each participant laid on a bed with shoulders abducted to 90°, elbows bent at 90° to the upper arms, and forearms in a neutral position. Each participant then rotated the arms toward the head (for ER) and the feet (for IR) until the arms could not move further, at which point the axis of the goniometer was placed on the olecranon. The fixed arm of the goniometer was perpendicular to the ground, and its mobile arm was parallel to the longitudinal axis of the ulna. The recorded angles between the two arms of the goniometer during ER and IR were the ROM for shoulder ER and IR, respectively. In the shoulder HA test, the same starting position as that for measuring ROM of shoulder rotation was used. Each participant then adducted the arms across the chest to reach the opposite shoulder. The axis of the goniometer was placed on the participant’s acromion with its fixed arm parallel to his or her shoulder, and the goniometer’s mobile arm was parallel to the longitudinal axis of the humerus. The angle recorded between the two arms was the ROM for shoulder HA [21]. These angles were measured to assess the flexibility of the posterior shoulder, with a greater angle indicating more severe posterior shoulder tightness.

A digital handheld dynamometer (microFET 2 Muscle Testing Dynamometer, Hoggan Scientific LLC, Salt Lake City, UT, USA) was used to measure shoulder muscle strength, measured for shoulder IR, ER, and HA. For the internal and external rotator muscle strength tests [22], each participant lay in a prone position with shoulders abducted to 90° and elbows bent by 90°. The researcher then placed the dynamometer on one of the participant’s wrists, and the participant was required to rotate his or her arms toward the feet (for IR) and head (for ER) with maximum strength and a forced contraction of 5 s. The average muscle strength over 5 s was recorded. The data acquired by the dynamometer during IR and ER were recorded as internal and external rotator muscle strengths, respectively. For the horizontal abductor muscle strength test, each participant laid in a prone position with shoulders abducted to 90° and elbows bent by 90°. The researcher then placed a dynamometer behind the participant’s distal upper arm above the elbow joint. The participant was required to raise his or her elbows toward the ceiling with maximum strength and a forced contraction of 5 s. The average strength over 5 s was recorded. The data acquired by the dynamometer were recorded as the shoulder’s horizontal abductor muscle strength.

The shoulder ROM and strength measurements were both performed by a senior physical therapist with a 16 years of experience. Before the formal study, the test-retest reliability of the shoulder ROM and strength measurements was assessed, and the results indicated high reliability (shoulder ROM measurements: intra-class correlation coefficient (ICC) = 0.837–0.952; shoulder muscle strength measurements: ICC = 0.837–0.976).

**Figure 2:**
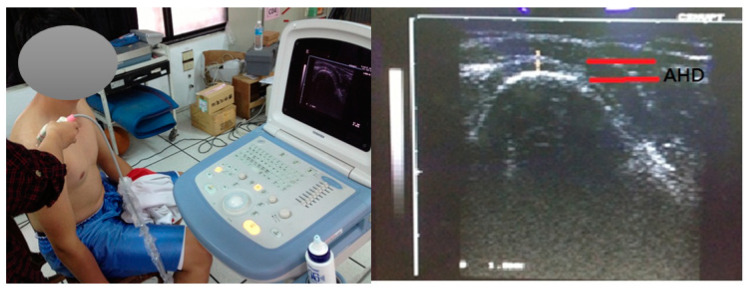
Sonographic images showing a measurement of the sub-acromial space. AHD: acromion to humeral head distance.

A musculoskeletal ultrasound diagnostic instrument (Diagnostic Ultrasound System, Famio 5, Toshiba, Japan) with a 5–10 MHZ linear transducer was used to evaluate the distance between the acromion and humeral head (AHD). The AHD measurement (Figure 2) involved each participant adopting a sitting position with the shoulders relaxed and naturally drooped. As taping was applied to the posterior and lateral parts of the shoulder, the AHD was measured from the anterior part of the shoulder [23]. During scanning, each participant had a shoulder abduction of 0°, and the transducer was parallel to the coronal plane. Before the formal study, the test-retest reliability of the AHD was carried out, and the results indicated high reliability (ICC = 0.983).

### 2.4. KT Application Procedure

**Figure 3:**
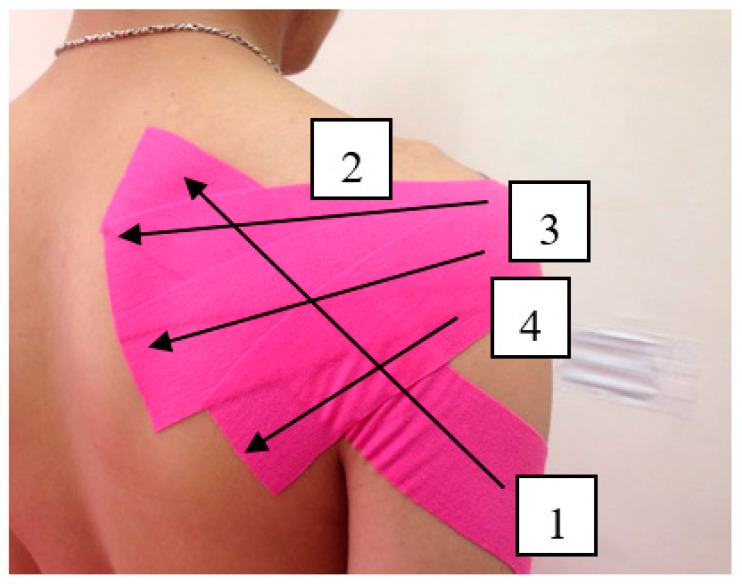
Kinesio taping applied on posterior shoulder. 1: posterior deltoid fibers; 2: upper infraspinatus fibers; 3: infraspinatus lower fibers; 4: teres minor.

A Kinesio tape (Kinesio Holding Company, Albuquerque, NM, USA; 4 m × 5 cm) was used for taping. In total, four strips of tape were used on the posterior deltoid fibers, infraspinatus, and teres minor. Each participant sat down, and taping was applied from the endpoints to the starting points of the relevant muscles to help the muscles relax. Muscle length was first measured, after which tape was cut into I-shaped strips of the corresponding lengths. No tensile force was applied during the tape application (Figure 3). The control group did not receive any tape application or stretching.

### 2.5. Sleeping Stretch Method

Each participant lay on the side of the dominant shoulder, the shoulders abducted to 90°, and elbows bent by 90°. The scapula was fixed using the participant’s body weight to avoid movement of the scapula. Each participant was required to grab the wrist of his or her dominant side with the non-dominant hand and slowly press the wrists in the direction of IR until feeling that the shoulder tissue was being pulled without any pain. This stretch was repeated five times, each lasting 30 s [5].

### 2.6. Statistical Analysis

The Statistical Package for Social Sciences software (SPSS 20, SPSS Inc., Chicago, IL, USA) was used for statistical analysis. The normality of the variables was evaluated with the Shapiro-Wilk test. The participants’ demographic information was used for the one-way analysis of variance (ANOVA) to analyze the level of heterogeneity among the groups. Subsequently, repeated measures two-factor ANOVA was conducted for the pre-test, post-test, and statistical analyses for the three groups (KT, SS, and control group). Post hoc analysis with Bonferroni correction was performed in the case of significant ANOVA findings for multiple comparisons between variables. All data are presented as the means and standard deviations for descriptive statistics, with the level of statistical significance set at α = 0.05.

## 3. Results

This study recruited 31 participants, each of the 31 participants was randomly assigned to the KT (N = 11), SS (N = 10), or control group (N = 10). The results showed no significant differences in terms of demographic information, indicating high homogeneity among the three groups (KT group: height 175.55 ± 3.93 cm, weight 73.64 ± 8.82 kg, age 20.36 ± 1.91 years; SS group: height 176.30 ± 5.77 cm, weight 79.10 ± 13.63 kg, age 20.50 ± 1.18 years; control group: height 180.50 ± 6.22 cm, weight 85.40 ± 10.69 kg, age 20.90 ± 1.45 years; *p* > 0.05). In the test groups, there were significant changes between the pretest and post-test values in relation to the measurement of shoulder ROM (IR: F = 11.872, *p* < 0.001, η^2^ = 0.459; ER: F = 6.379, *p* = 0.005, η^2^ = 0.313; HA: F = 7.336, *p* = 0.003, η^2^ = 0.344; Total rotation: F = 12.617, *p* < 0.001, η^2^ = 0.474). In the post-test measurements, ROM in relation to shoulder IR, HA, and total rotation in the KT group and SS group increased, whereas shoulder ER exhibited reduced ROM in these two groups.

For the shoulder muscle strength test, only shoulder external rotators showed a significant difference in muscle strength post-test (F = 10.494, *p* < 0.001, η^2^ = 0.428). Specifically, the KT group exhibited increased muscle strength post-test, whereas the SS group had reduced muscle strength; muscle strength remained unchanged in the control group. Furthermore, the sub-acromial space exhibited no significant changes after the intervention and among the groups (F = 0.809, *p* = 0.462, η^2^ = 0.087) (Table 1 and Table 2).

## 4. Discussion

The results revealed that KT and SS both significantly improved shoulder ROM in terms of shoulder IR, HA, and total rotation. However, the ROM for shoulder ER declined slightly after KT treatment, whereas it increased slightly after SS. For the shoulder muscle strength test, external rotator muscle strength was significantly improved after KT, whereas SS significantly reduced external rotator muscle strength; no significant changes were observed in the other muscle strength tests.

The present study verified the effect of SS while testing an alternative method of relieving posterior shoulder tightness by applying KT. The results revealed that KT increased the ROM in relation to shoulder IR, HA, and total rotation. Therefore, SS and KT are both helpful for pitchers with GIRD. However, recent studies on stretching exercises have determined that performing static stretching before exercise reduces muscle strength and performance [3,9,10,24]; therefore, the shoulder muscle strength test was conducted in the present study to gain an understanding of muscle strength changes.

In this study, the KT group had increased ER strength after the application of Kinesio tape, whereas ER strength declined in the SS group following stretching. Therefore, similarly to the results of previous studies on stretching, SS reduced the strength of the ER muscles, whereas KT improved the strength of the ER muscles. However, previous studies on the effects of KT on muscle function have drawn no clear conclusions on muscle strength or power [17] with most scholars believing that the effects of KT can improve muscle recruitment, thereby affecting muscle contraction timing and coordination between muscles [25,26,27]. This assertion contradicts the findings of the present study, which demonstrated that KT could improve muscle strength in two manners. First, tight muscles can be released through KT because the tape’s elastic material increases flexibility and improves ROM, thereby enabling the tissue to exert force [15]. Second, regarding the elastic effect of KT, Bravi and coworkers [28] discovered that elastic KT could improve the timing variability of repetitive rhythmic movement, which may be a result of KT providing proprioceptive feedback from the skin’s surface. This facilitates adjustment of the rhythmic movement process and further improves the coordination of muscle contractions, thereby may possibly affecting muscle strength output.

The results of the present study revealed that neither KT nor SS affected sub-acromial space, despite most related studies having focused on evaluating sub-acromial space in patients with shoulder impingement syndrome after applying taping or stretching [11,12,14,29]. Although such studies have noted that taping and stretching can improve sub-acromial space, the present study observed that taping did not affect sub-acromial space. This may be due to two possible reasons. First, the participants in this study had GIRD but had not developed shoulder impingement. This observation differed from those of previous studies that recruited participants with shoulder impingement or shoulder injuries. Second, previous uses of ultrasound to measure the sub-acromial space have involved scanning of the posterior shoulder region and lateral deltoid fibers [11]. However, the application of tape to the posterior deltoid fibers in this study rendered evaluation through ultrasound scanning of the posterior shoulder region impossible. Therefore, scanning was performed on the anterior deltoid fibers. The different scanning positions may have contributed to the difference in results for this study compared to other studies. This is one limitation of this study. However, McCreesh et al. [23] noted the reliability of conducting an ultrasound scan of the sub-acromial space from the anterior part of the shoulder. The inter-participant reliability and intra-participant reliability were both 0.92–0.98. In the present study, an ultrasound scan was performed by one researcher, who achieved test-retest reliability of 0.983, indicating high reliability. Thus, ultrasound scanning of the sub-acromial space can also be conducted on the anterior deltoid. Another limitation is that this study mainly explored the effects of KT and SS on GIRD. However, in addition to posterior shoulder tightness, another cause of GIRD is long-term repetitive overhead movements resulting in humeral torsion—a problem of bone adaptation as opposed to soft tissue changes. KT and SS are treatments for soft tissue, and thus cannot relieve humeral torsion. Further research can identify other alternative ways to solve the problems due to repeated throwing, which results in problems related to bone adaptation or humeral torsion in GIRD pitchers.

## 5. Conclusions

Pitchers with GIRD are recommended to apply KT before competition or training to increase the shoulder’s ROM; the SS should be performed after competition or training to mitigate the impact of stretching on muscle strength and facilitate shoulder mobility.

## Figures and Tables

**Table 1:** Results for shoulder joint ROM, muscle strength, and sub-acromial space in the Kinesio taping (KT) group, sleeper stretching (SS) group, and control group.

	KT Group		SS Group		Control Group	
	Pre	Post	Pre	Post	Pre	Post
**Shoulder ROM**						
IR, degree	59.19 ± 7.10 *	68.25 ± 4.37 *	57.46 ± 9.05 *	70.94 ± 10.93 *	52.02 ± 11.92	52.23 ± 12.41
ER, degree	101.95 ± 11.00	98.75 ± 10.71	98.29 ± 11.03	101.30 ± 10.63	104.96 ± 14.11	103.13 ± 13.89
HA, degree	112.66 ± 10.67 *	124.31 ± 9.94 *	115.46 ± 9.54 *	123.46 ± 11.12 *	118.69 ± 19.12	121.13 ± 16.33
Total rotation, degree	161.12 ± 14.98 *	166.95 ± 13.70 *	155.77 ± 9.30 *	172.24 ± 8.13 *	156.99 ± 18.55	155.36 ± 16.33
**Shoulder strength**						
IR, lb	37.32 ± 8.91	38.67 ± 9.52	34.95 ± 7.98	35.22 ± 10.21	34.14 ± 9.67	35.33 ± 9.86
ER, lb	30.48 ± 8.89	34.28 ± 9.94	33.18 ± 9.20	30.38 ± 9.75	31.21 ± 9.62	32.84 ± 9.11
HAbd, lb	36.18 ± 7.06	38.54 ± 9.10	37.08 ± 11.41	38.30 ± 10.27	40.96 ± 9.79	37.22 ± 7.93
**Sub-acromial Space**						
AHD, mm	11.77 ± 1.95	10.57 ± 0.94	10.37 ± 4.09	10.90 ± 2.42	7.58 ± 3.37	7.71 ± 3.70

ROM: range of motion; IR: internal rotation; ER: external rotation; HA: horizontal adduction; HAbd: horizontal abduction; AHD: the distance between the acromion and humeral head from anterior shoulder view. *: There were significant differences between the pre-test and post-test values in KT and SS groups.

**Table 2:** Statistical analysis results for shoulder joint ROM, muscle strength, and sub-acromial space in the Kinesio taping (KT) group, sleeper stretching (SS) group, and control group.

	Within-Subject (Pre-Post)			Between-Subject (Group)			Interaction (Group x Pre-Post)			
	F_(1,28)_	*p*	Partial Eta Squared	F_(2,31)_	*p*	Partial Eta Squared	F_(2,31)_	*p*	Partial Eta Squared	Power
**Shoulder ROM**										
IR	46.161	0.000	0.622	5.737	0.008	0.291	11.872	0.000	0.459	0.990
ER	0.817	0.374	0.028	0.389	0.681	0.027	6.379	0.005	0.313	0.867
HA	54.965	0.000	0.663	0.034	0.967	0.002	7.336	0.003	0.344	0.911
Total rotation	22.395	0.000	0.444	1.160	0.328	0.077	12.617	0.000	0.474	0.993
**Shoulder Muscle Strength**										
IR	0.940	0.341	0.032	0.422	0.660	0.029	0.121	0.887	0.009	0.067
ER	2.114	0.157	0.070	0.011	0.989	0.001	10.494	0.000	0.428	0.979
HAbd	0.001	0.971	0.000	0.121	0.887	0.242	1.731	0.196	0.110	0.332
**Sub-acromial Space**										
AHD	0.106	0.749	0.006	2.905	0.082	0.255	0.809	0.462	0.087	0.165

ROM: range of motion; IR: internal rotation; ER: external rotation; HA: horizontal adduction; HAbd: horizontal abduction; AHD: the distance between the acromion and humeral head from anterior shoulder view.

## Data Availability

The data that support the findings of this study are available on request from the corresponding author. The data are not publicly available due to privacy or ethical restrictions.
